# Inter- and intra-species conversion efficacies of Norwegian prion isolates estimated by serial protein misfolding cyclic amplification

**DOI:** 10.1186/s13567-023-01220-7

**Published:** 2023-09-29

**Authors:** Erez Harpaz, Tram Thu Vuong, Linh Tran, Michael Andreas Tranulis, Sylvie L. Benestad, Cecilie Ersdal

**Affiliations:** 1 Department of Production Animal Clinical Sciences, Faculty of Veterinary Medicine, Norwegian University of Life Sciences, Svebastadveien 112, 4325 Sandnes, Norway; 2 Department of Biohazard and Pathology, Norwegian Veterinary Institute, P.O. box 64, 1431 Ås, Norway; 3 Department of Preclinical Sciences and Pathology, Faculty of Veterinary Medicine, Norwegian University of Life Sciences, Universitetstunet 3, 1433 Ås, Norway

**Keywords:** Cervids, CWD, Norway, PMCA, prion, *PRNP*, reindeer, scrapie, species-barrier, spillover

## Abstract

Prion diseases, including chronic wasting disease (CWD) in cervids, are fatal neurodegenerative disorders caused by the misfolding of cellular prion proteins. CWD is known to spread among captive and free-ranging deer in North America. In 2016, an outbreak of contagious CWD was detected among wild reindeer in Norway, marking the first occurrence of the disease in Europe. Additionally, new sporadic forms of CWD have been discovered in red deer in Norway and moose in Fennoscandia. We used serial protein misfolding cyclic amplification to study the ability of Norwegian prion isolates from reindeer, red deer, and moose (two isolates), as well as experimental classical scrapie from sheep, to convert a panel of 16 brain homogenates (substrates) from six different species with various prion protein genotypes. The reindeer CWD isolate successfully converted substrates from all species except goats. The red deer isolate failed to convert sheep and goat substrates but exhibited amplification in all cervid substrates. The two moose isolates demonstrated lower conversion efficacies. The wild type isolate propagated in all moose substrates and in the wild type red deer substrate, while the other isolate only converted two of the moose substrates. The experimental classical scrapie isolate was successfully propagated in substrates from all species tested. Thus, reindeer CWD and classical sheep scrapie isolates were similarly propagated in substrates from different species, suggesting the potential for spillover of these contagious diseases. Furthermore, the roe deer substrate supported conversion of three isolates suggesting that this species may be vulnerable to prion disease.

## Introduction

Chronic wasting disease (CWD) is a neurodegenerative disorder affecting several species of cervids [1]. The disease was first described in 1967 in a captive mule deer in Colorado, USA, and characterized later as a transmissible spongiform encephalopathy (TSE) [2]. The cause of these disorders is an alteration in the three-dimensional structure of the normal cellular prion protein (PrP^C^), which is converted into a beta-sheet-rich, misfolded prion protein (PrP^Sc^) and further amplified in a templated manner [3]. There are several known TSEs in both humans and animals, which, due to their shared etiology, are commonly referred to as prion diseases. In the contagious forms of prion diseases, classical scrapie in small ruminants and classical CWD in cervids, disease associated prions can propagate in lymphoid tissues, and in later stages will disseminate in the central nervous system (CNS) where they cause spongiform changes [4, 5]. All prion diseases are fatal and end with the death of the host [6].

In 2016, the first reported classical CWD case in Europe was found in a free-ranging reindeer (*Rangifer tarandus tarandus*) in the Nordfjella reindeer area in the south of Norway [7]. This was also the first case of natural CWD infection in reindeer, although reindeer have been experimentally infected with CWD earlier [8, 9]. Following this event, due to intensifying monitoring, two additional cases of classical CWD were detected in reindeer in 2019 and 2022 in a previously unaffected region, the Hardangervidda plateau. In addition, sporadic CWD cases were found in moose (*Alces alces*) [10] and red deer (*Cervus elaphus elaphus*) [11] in several Norwegian counties. In total, 21 reindeer, 11 moose, and three red deer have to date been diagnosed with CWD in Norway [12], leaving roe deer (*Capreolus capreolus*) as the only native cervid species in Norway with no documented CWD cases. The CWD-positive reindeer cases consisted predominantly of young adults and adults with age range of 1–8 years. In 52.4% of the cases, PrP^Sc^ was found in both the lymph nodes and the CNS, while in the remaining of the animals, only the lymph node tested positive for PrP^Sc^ [12–14]. In contrast, Norwegian cases of moose and red deer with available age data exhibited a notably higher mean age of 14.9 years, with prions exclusively detectable in the CNS [10, 11, 13]. Besides the Norwegian cases, Sweden has reported four cases of moose CWD, while Finland has reported three cases [15, 16]. Detailed analysis revealed significant differences in disease characteristics between moose and reindeer cases, as well as between moose cases, all distinct from the well-established North American CWD isolates [10, 15, 17], including their potential for interspecies transmission [17, 18].

The “species barrier” refers to the inefficiency of a prion disease to transmit and establish in a different species [19, 20]. Phylogenetically related species may transmit prion diseases more easily such as scrapie between goats and sheep and CWD between different cervids [21]. However, prion diseases can sometimes be transmitted to distantly related species and may also have zoonotic potential, as exemplified by the tragic emergence of variant Creutzfeldt-Jakob disease (vCJD) in humans, which derived from bovine spongiform encephalopathy (BSE) and overcame the species barrier [22, 23]. Susceptibility to most prion diseases is influenced by prion protein gene (*PRNP*) polymorphisms which, in contagious diseases, can determine the fate of the prion within the host following uptake. Similarly, *PRNP* variation will influence the likelihood of spontaneous conversion of PrP^C^ in the brain [24–27].

The presence of multiple prion strains, even within a single host, may account for the manifestation of varied disease phenotypes, despite the host's PrP^C^ having a similar primary structure [28]. According to the conformation selection theory, a “strain” is, in fact, a group of prions with diverse three-dimensional shapes and biomolecular characteristics. Some of these conformers may be compatible with the target species PrP^C^, which may explain how certain strains, or isolates, can cross the species barrier and cause disease. On the other hand, if none of the prion conformers within the host are compatible with the target species PrP^C^, transmission will not occur [29].

Norway has abundant wild and semi-domesticated reindeer as well as free-roaming moose, roe deer, and red deer. These cervids exhibit migratory behavior [30], making their movements and interactions with other species difficult to control. During summers, sheep are released to graze in mountain pastures, and as demonstrated in Nordfjella, sheep and reindeer spatial overlapping exists, hence the potential risk of CWD spillover to other species [31, 32].

Several species have been experimentally inoculated with different North American CWD isolates [33–37], but inoculation studies using Norwegian CWD isolates are sparse, and so far, only experiments in bank vole and genetically modified mice have been published [17, 38, 39].

Protein misfolding cyclic amplification (PMCA) can be employed to offer in-vitro data on prion replication when inoculation studies are missing. This technique involves repeated cycles of incubation and sonication, where a PrP^Sc^ seed, or isolate, is added to a PrP^C^-rich substrate (usually homogenized normal brain) to start prion conversion. In the sonication phase, prion aggregates are thought to be fragmented, exposing more reactive sites, and increasing the seeding conversion activity in the subsequent incubation phase. These incubation-sonication cycles continue until the PrP^C^ content in the substrate is exhausted, usually after 48 h [40]. An aliquot of the product can then be added to a fresh substrate which in turn can be subjected to a new round of cyclic sonication and incubation called serial, or sPMCA.

The objective of this study was to assess the conversion potential of various Norwegian CWD isolates towards several cervid species as well as domestic small ruminants. We utilized prion isolates from CWD-affected reindeer, moose, and red deer and an experimental classical sheep scrapie isolate, as seeds in sPMCA reactions. As substrates, we used brain homogenates from the four cervid species native to Norway, as well as sheep and goats with different *PRNP* genotypes.

## Materials and methods

### Source of brain substrates

Reindeer brains, except for the one with the wild type *PRNP*, were collected during the slaughter of semi-domesticated reindeer from Nordland County in northern Norway. The wild type brain was collected during the slaughter of semi-domesticated reindeer from the Filefjell reindeer herding unit. The brains of the other cervid species were acquired during the autumn 2020 hunting season. The cervid brains were frozen at −20 °C as soon as possible after collection and later transferred to a −70 °C freezer. Fresh, healthy sheep brains were collected during necropsies performed at the Section for Small Ruminant Research and Herd Health, Faculty of Veterinary Medicine, Norwegian University of Life Sciences, or immediately after slaughter at the Nortura Forus abattoir. These brains were frozen at −70 °C immediately after sampling. The goat brains were obtained from a previous experiment [41]. All brain substrates in the study were tested negative for PrP^Sc^ by Enzyme-Linked Immunosorbent Assay (HerdCheck BSE/scrapie Ag test, IDEXX). The brain substrates used in the experiment are summarized in Table 1.

**Table 1:** **Brains used as sPMCA substrates and their respective** ***PRNP* genotypes**.

Species	*PRNP* genotype
Reindeer	S_225_/S_225_ (wild type)
	Y_225_/Y_225_
	D_176_/D_176_
	M_2_S_129_M_169_/M_2_S_129_M_169_
	S_225_/Y_225_
Red deer	Q_226_/Q_226_ (wild type)
	E_226_/E_226_
Roe deer	Q_226_/Q_226_ (wild type)
Moose	K_109_/K_109_ (wild type)
	Q_109_/Q_109_
	Q_109_/K_109_
Sheep	A_136_R_154_Q_171_/A_136_R_154_Q_171_ (wild type)
	V_136_R_154_Q_171_/V_136_R_154_Q_171_
	A_136_R_154_R_171_/A_136_R_154_R_171_
Goat	Q_220_/L_220_
	Ter_32_/Ter_32_ (no PrP^C^ expression)

### *PRNP* genotyping

Deoxyribonucleic acid (DNA) was extracted from either blood or brain tissue using the DNeasy Blood & Tissue Kit (Qiagen GmbH) following the manufacturer's instructions. Cervid *PRNP* genotypes were determined by sequencing the plus strand of the *PRNP* open reading frame after PCR amplification using the following primers: forward 5′-ATTTTGCAGATAAGTCATCATG-3′ and reverse 5′-AGAAGATAATGAAAACAGGAAG-3′. The PCR reaction included 5 µL of genomic DNA as a template, 25 µL of DreamTaq Green PCR Master Mix (Thermo Fisher Scientific), 2.5 µL of forward and reverse primers (10 µM) (Thermo Fisher Scientific), and nuclease-free water to reach a final volume of 50 µL. The PCR amplification consisted of an initial cycle at 95 °C for 3 min, followed by 36 cycles at 95 °C for 30 s, 56 °C for 30 s, and 72 °C for 45 s, and a final extension at 72 °C for 7 min. The coding region of the goat *PRNP* was analyzed similarly using the following primers: forward 5′-GATGCCACTGCTATGCAGTCAT-3′ and reverse 5′-AAAACAGGAAGGTTGCCCCT-3′. For sheep, selective *PRNP* genotyping at amino acid positions 136, 154, and 171 was performed by a commercial laboratory (Eurofins Genomic GmbH). The accession numbers for the wild type *PRNP’s* are ACT87519.1 for sheep, MN784961.1 for reindeer, JQ290077.1 for moose, MK103027.1 for red deer, and MK103016.1 for roe deer.

### Prion isolates

The isolates used as seeds are listed in Table 2. The reindeer CWD isolate originates from the Nordfjella outbreak and has not been characterized earlier. The moose and red deer isolates were identified through the national surveillance program for CWD. The wild type moose isolate, ID CD11399 [10, 17, 18, 39] and red deer isolate ID CD14051 [18] have been previously characterized. The sheep scrapie isolate originates from an experimental study [42].

**Table 2:** **Prion isolates used as seed in the sPMCA experiments**.

Species	ID	*PRNP* genotype	Type of isolate	Tissue
Reindeer	CD24089	S_225_/S_225_ (wild type)	Contagious	Lymph node
Moose	CD11399	K_109_/K_109_ (wild type)	Sporadic	Brain
Moose	CD24854	Q_109_/Q_109_	Sporadic	Brain
Red deer	CD14051	E_226_/E_226_	Sporadic	Brain
Sheep	7233	V_136_R_154_Q_171_/V_136_R_154_Q_171_	Contagious	Brain

### Serial protein misfolding cyclic amplification procedure

To prepare the substrates, brain tissues were homogenized using a Potter–Elvehjem tissue grinder with a conversion buffer consisting of 1X PBS, 150 mM NaCl, 1% Triton X-100, and a protease inhibitor cocktail (Roche) in a 1/10 ratio. The homogenates were then centrifuged at 800 g at 4 °C for 2 min. After centrifugation, the supernatant was transferred to 1 mL Eppendorf tubes and frozen at −70 °C. Before starting the PMCA procedure, the substrate tubes were thawed, and 100 µg/mL heparin and 0.05% digitonin were added. A mixture of 10 µL of seed and 90 µL of substrate was added to a PCR tube containing two Teflon beads and then placed in a sonicator horn connected to a Q700 sonicator (Qsonica). The mixture underwent 48 h of sonication-incubation cycles, which involved 30 s of sonication at 230-250W and 29 min and 30 s of incubation at 37 °C. After each 48-h cycle, the procedure was repeated using 10 µL of the product added to 90 µL of fresh substrate. Each 48-h sonication-incubation cycle was considered one round of PMCA. For the PMCA experiments, we included negative controls consisting of normal brain homogenate (substrate) seeded with PrP^Sc^-negative brain or lymph node (reindeer) homogenate. The source of the PrP^Sc^ negative seeds corresponded to the species from which the PrP^Sc^-positive seed used in that experiment originated. In addition, at least one tube with non-seeded brain homogenate was included in each experiment to monitor for potential cross-contamination.

### Proteinase K digestion and western blotting

Western blot was performed on the original isolates, some of the substrates, and the PMCA products using the TeSeE Western Blot kit (Bio-Rad) following the manufacturer’s instructions. The tested PMCA products and the isolates were subjected to a one-hour digestion with 100 µg/mL proteinase K (PK) (Sigma-Aldrich) at 37 °C. This step was omitted for the cervid substrates to detect the host PrP^C^. The reaction was stopped by incubating the mixture with Laemmli sample buffer (Bio-Rad) for five minutes at 100 °C. The samples were then loaded onto Sodium Dodecyl Sulfate–Polyacrylamide gel (NuPAGE 12% Bis–Tris protein gels, Thermo-Fisher) and electrophoresed at 100 V for 55 min. The proteins were subsequently transferred to a polyvinylidene difluoride membrane using a trans-blot turbo system (Bio-Rad), and the membrane was then subjected for immunodetection. The commercial kit included the primary monoclonal antibody (mAb), Sha31, which recognizes the core epitope 148YEDRYYRE155. Additionally, the mAb P4 (Labolytic), which identifies the N-terminal epitope 93WGQGGSH99 (ovine numbering for both antibodies), was used. The results were visualized using Azure c280 (Azure Biosystems). Brains from a scrapie-positive sheep, either alone or together with a moose CWD isolate, were utilized as positive controls.

## Results

### sPMCA using normal brain homogenate as substrate

**Figure 1:**
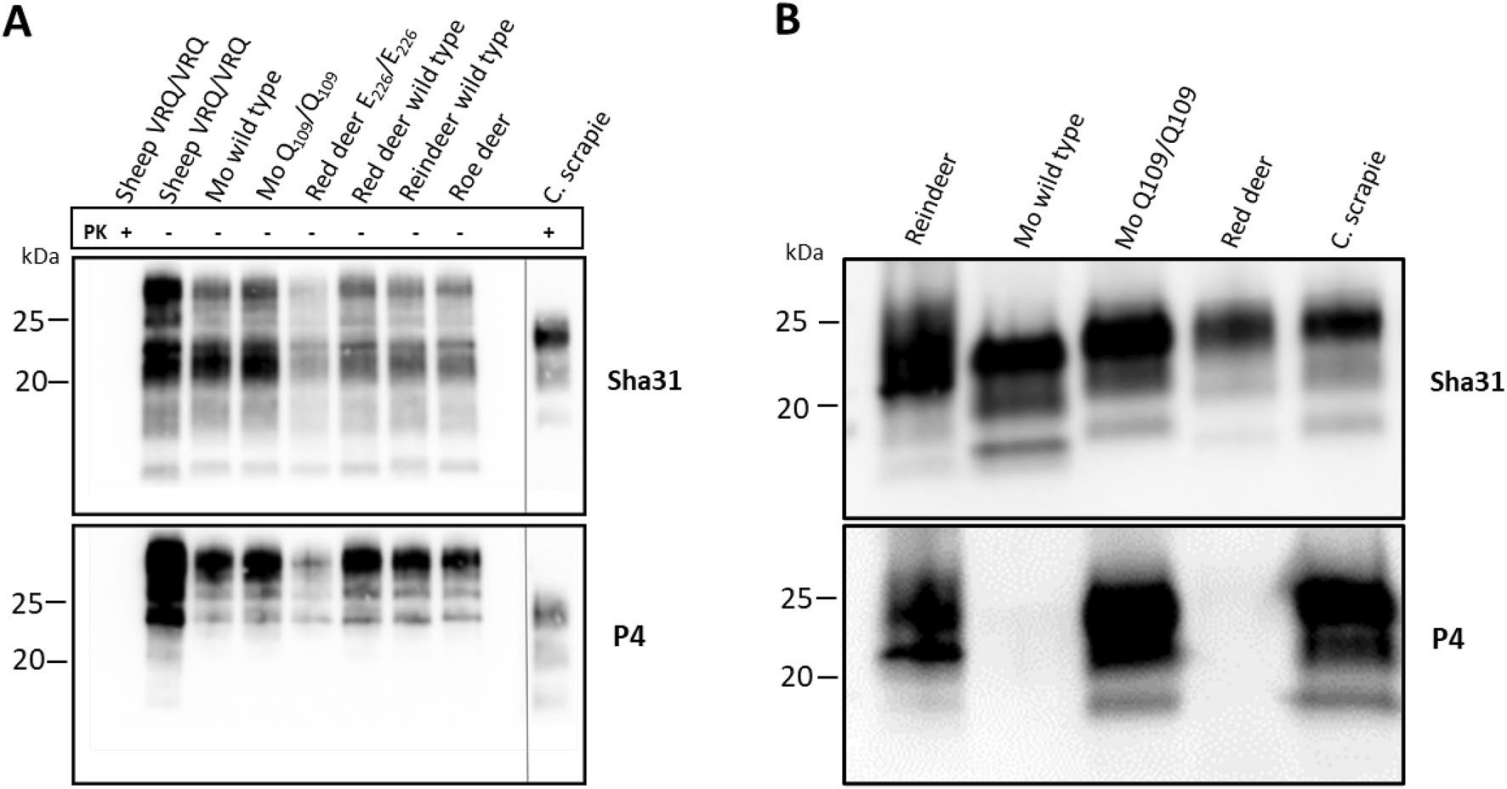
**Western blot analysis of cervid substrates and prion isolates for PrP content with monoclonal antibodies Sha31 and P4**. **A** Both Sha31 and P4 effectively identified PrP^C^ in brain homogenates of all cervid species in the study. Three control samples were included: normal VRQ/VRQ sheep homogenate with and without proteinase K (PK) treatment and a VRQ/VRQ classical scrapie with prion banding pattern after PK treatment.** B** The same antibodies were used to visualize the original prion isolates included in the study. The P4 mAb did not detect the wild type moose and red deer isolates. The two contagious isolates, but also the sporadic moose Q_109_/Q_109_ isolate kept the P4 epitope. All samples were subjected to PK treatment. *VRQ* V_136_R_154_Q_171_, *Mo* moose, *C. scrapie* classical scrapie, *kDa* molecular weight in kilodalton.

**Figure 2:**
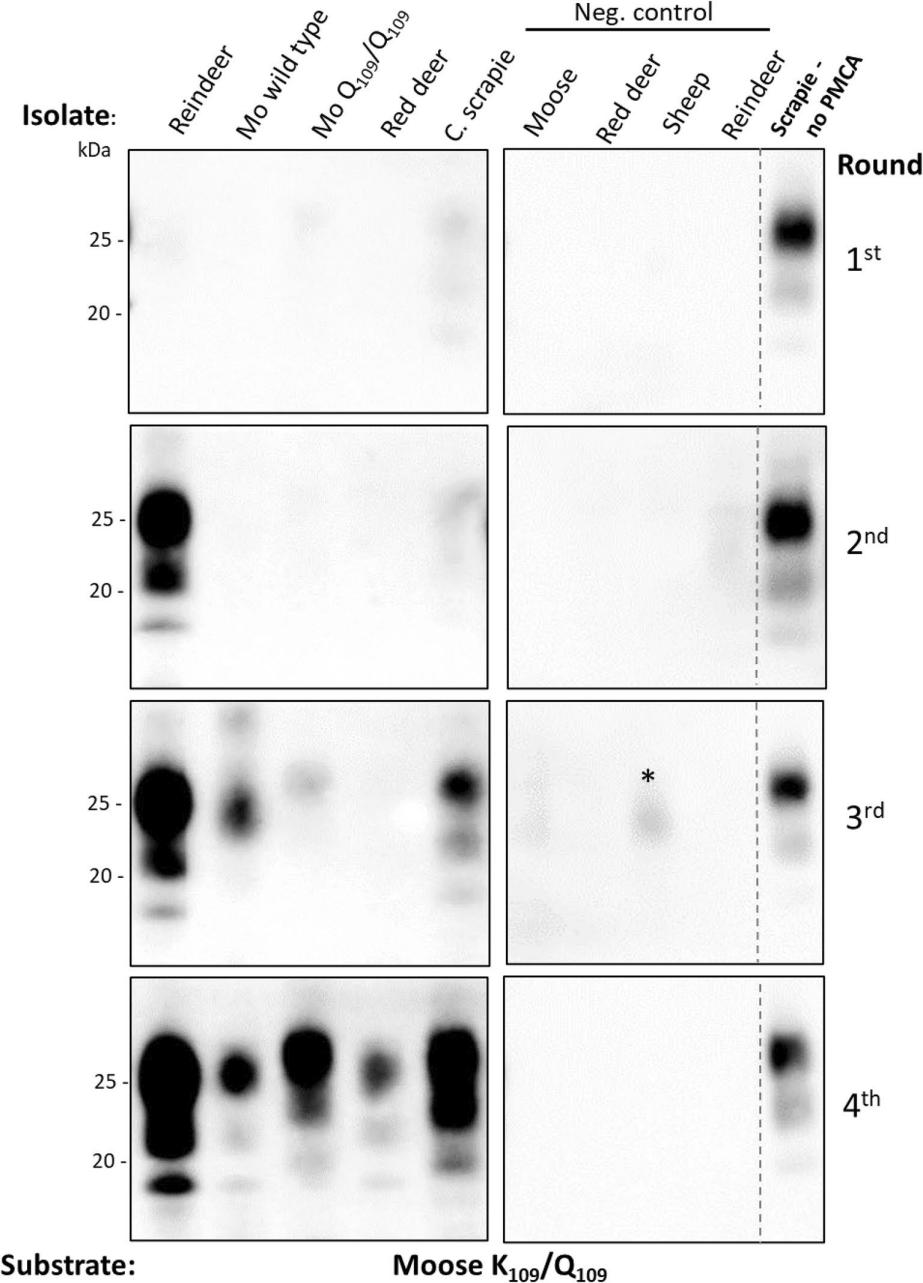
**Representative western blots showing sPMCA of Norwegian CWD and experimental classical scrapie isolates in the moose brain substrate K**_**109**_**/Q**_**109**_ **variant**. Cervid CWD and experimental classical scrapie isolates were subjected to sPMCA using moose K_109_/Q_109_ brain as substrate**.** After each PMCA round, the amplified products were digested with proteinase K and analyzed by western blotting. After four rounds of PMCA, all the isolates were amplified and characteristic prion bands could be seen in the western blots. *Mo* moose, *C. scrapie* experimental classical scrapie, * Unspecific band, *kDa* molecular weight in kilodalton.

**Figure 3:**
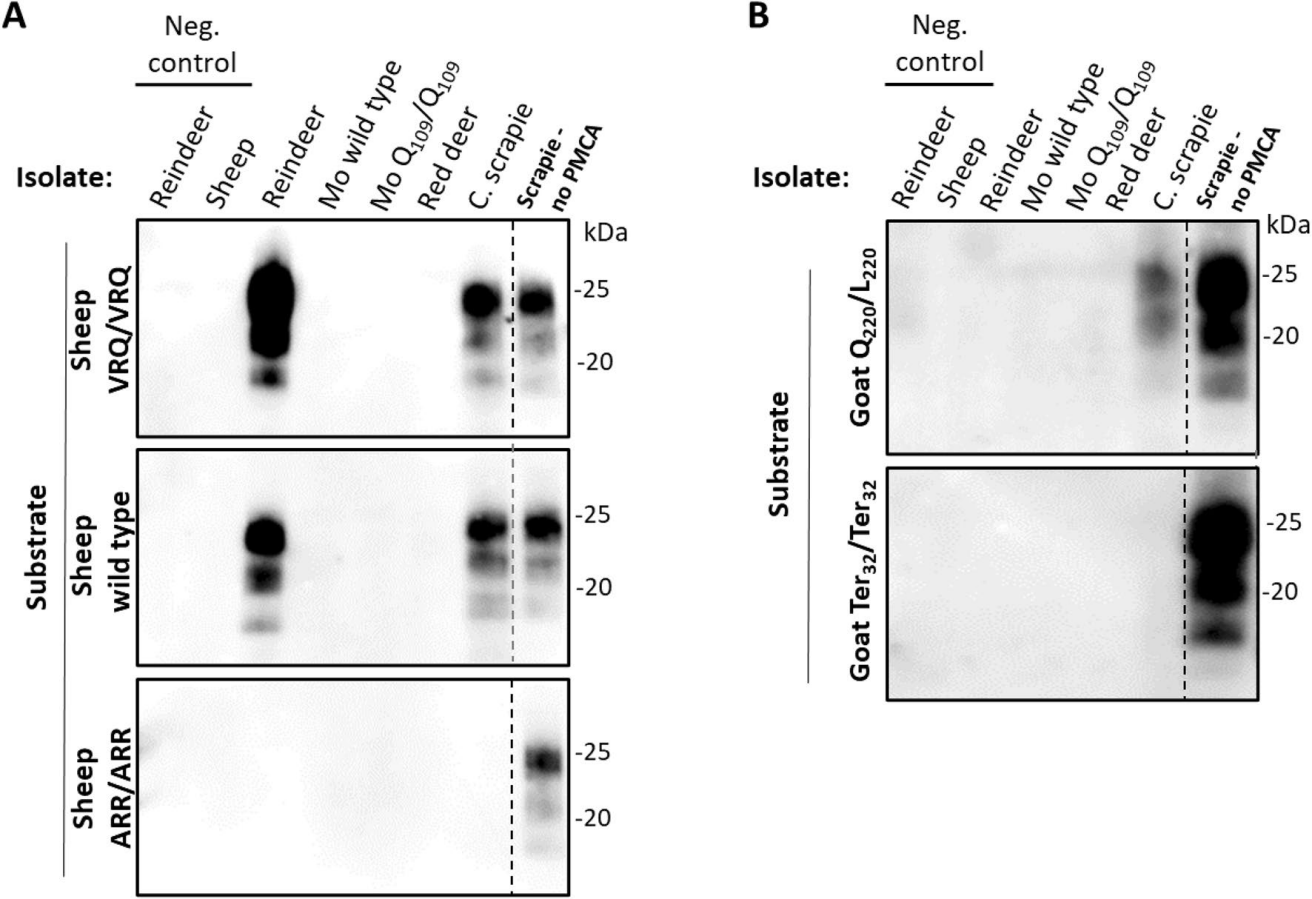
**Amplification of Norwegian CWD and experimental classical scrapie isolates in sheep and goat substrates after 8 rounds of PMCA**. **A** Only the reindeer and scrapie isolates were amplified in VRQ/VRQ and ARQ/ARQ sheep substrates. **B** Only the experimental classical scrapie isolate was amplified in goat Q_220_/L_220_ substrate. None of the isolates were amplified in the sheep brain with the most scrapie resistant genotype ARR/ARR and the goat Ter_32_/Ter_32_ substrate without expression of the cellular prion protein. *VRQ/VRQ* V_136_R_154_Q_171_/V_136_R_154_Q_171_, *ARR/ARR* A_136_R_154_R_171_/A_136_R_154_R_171_, *Mo* moose, *C. scrapie* experimental classical scrapie, *kDa* molecular weight in kilodalton.

We tested the ability of prion isolates from Norwegian cervids and sheep to be amplified in brain homogenates from different species with different *PRNP* genotypes. We used a total of five different isolates as seeds, derived from four species: reindeer, moose (two isolates), red deer, and sheep (Table 2). As substrates, we utilized brain homogenate from six different species with different *PRNP* genotypes, totaling 16 brain homogenates (Table 1), some of which represented the wild type *PRNP* for the respective species. For each species, and when applicable, we employed substrates with varying susceptibility to prion disease based on current knowledge. We also performed western blotting on the cervid brain homogenates to confirm that P4 and Sha31 mAbs are suitable for detecting cervid PrP^C^. These antibodies target two different regions on the prion protein: Sha31 targets the core region, and P4 targets the N-terminal region. Both antibodies recognized PrP^C^ in all tested species (Figure 1A). We observed that the contagious isolates from reindeer CWD and experimental sheep scrapie exhibited strong conversion abilities (Figures 2 and 3, Table 3). Interestingly, the reindeer isolate only converted the wild type reindeer substrate and none of the other reindeer substrates. The red deer isolate also converted substrates from all cervids, but not the sheep and goat brains. The moose wild type isolate converted all moose substrates as well as the red deer wild type brain, while the moose Q_109_/Q_109_ isolate only converted two of the moose substrates. The roe deer substrate was converted by three out of five seeds, including both contagious CWD isolates. As expected, no conversion was observed in the scrapie resistant A_136_R_154_R_171_/A_136_R_154_R_171_ sheep substrate and the goat Ter_32_/Ter_32_ substrate without expression of cellular PrP (Figure 3). The complete results are presented in Table 3.

**Table 3:** **sPMCA conversion results**.

Brain substrate		Prion isolate				
Species	*PRNP*-genotype	sPMCA amplification				
		Reindeer CWD	Moose CWD		Red deer CWD	Sheep scrapie
		wt	wt	Q_109_/Q_109_	E_226_/E_226_	V_136_/V_136_
Reindeer	S_225_/S_225_ (wt)	+ + + +	−	−	+	+ + +
	S_225_/Y_225_	−	−	−	−	−
	Y_225_/Y_225_	−	−	NA	NA	NA
	D_176_/D_176_	−	−	NA	NA	NA
	M_2_S_129_M_169_/M_2_S_129_M_169_	−	−	NA	NA	NA
Moose	K_109_/K_109_ (wt)	+ + +	+ +	-	+ + +	+ + +
	Q_109_/Q_109_	+ + + +	+ + +	+ +	+ + +	+ + + +
	K_109_/Q_109_	+ + + +	+ + +	+ + +	+ + +	+ + +
Red deer	Q_226_/Q_226_ (wt)	+ + +	+ +	−	+ +	+ + + +
	E_226_/E_226_	+ + + +	−	−	+ +	+ + + +
Roe deer	Q_226_/Q_226_ (wt)	+ +	−	−	+ +	+ + +
Sheep	A_136_R_154_Q_171_/A_136_R_154_Q_171_ (wt)	+	−	−	−	+ + + +
	V_136_R_154_Q_171_/V_136_R_154_Q_171_	+	−	−	−	+ + +
	A_136_R_154_R_171_/A_136_R_154_R_171_	−	−	−	−	−
Goat	Q_220_/L_220_	−	−	−	−	+
	Ter_32_/Ter_32_	−	−	−	−	−

The grading indicates the PMCA round of first detectable amplification by western blot, following digestion with proteinase K (100 µg/mL final concentration): + , round 7–8; +  + , rounds 5–6; +  +  + , round 3–4; +  +  +  + , round 1–2;—– no amplification; *NA* not performed, *wt* wild type, *V*_*136*_*/V*_*136*_—V_136_ R_154_ Q_171_/V_136_ R_154_ Q_171_, *Ter*_*32/*_*Ter*_*32*_ no expression of PrP^C^.

### Biochemical property characterization of sPMCA products

Since the moose and red deer CWD isolates have different N-terminal PK-cleavage sites compared to the contagious prion isolates [10, 16], we wanted to further characterize the included isolates. The Sha31 antibody detects all PrP^Sc^ present, while the P4 antibody only recognizes products which retain this epitope of the N-terminal following PK treatment. Following PK digestion of the non-amplified isolates, both the wild type moose and the red deer isolates lost their P4 epitope, while the other isolates maintained it (Figure 1B).

**Figure 4:**
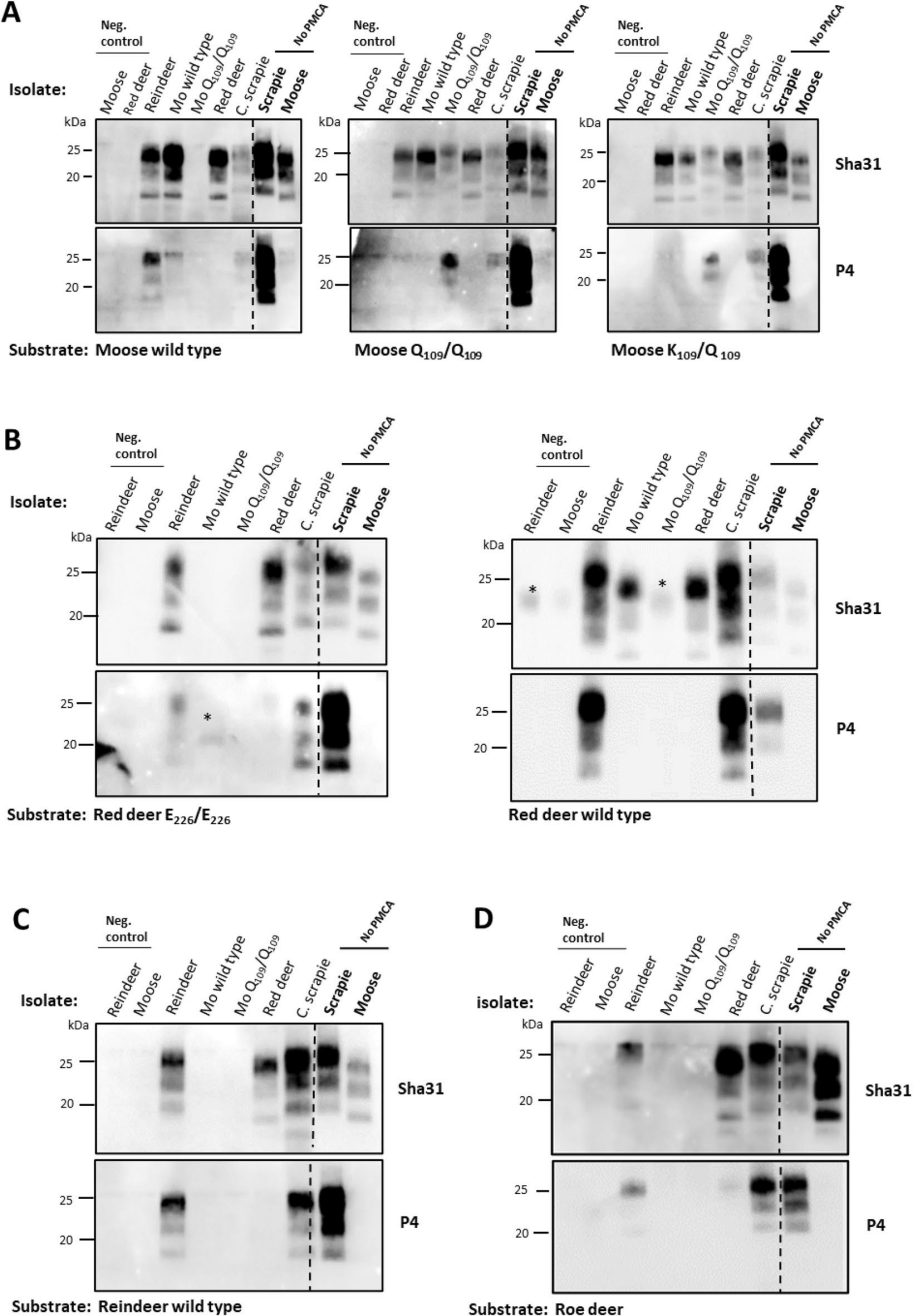
**Biochemical properties of the PMCA products by discriminating primary antibodies.** The Sha31 antibody, recognizing the core region and the P4 antibody targeting the N-terminal of the PrP^Sc^ were used to assess the biochemical properties of the amplified products in **A** moose, **B** red deer, **C** reindeer and **(D)** roe deer substrates. The amplified PrP^Sc^ exhibited properties similar to those of the original isolates except for the reindeer isolate that lost the P4 epitope in the moose Q_109_/Q_109_ and K_109_/Q_109_ substrates. * Unspecific band; *Mo* moose. *C. scrapie* experimental classical scrapie, *kDa* molecular weight in kilodalton.

We observed that the reindeer isolate lost the P4 epitope following amplification in the Q_109_/Q_109_ and the K_109_/Q_109_ moose substrates (Figure 4A), while the other contagious seed, the classical scrapie, kept its P4 epitope in all amplified substrates (Figures 4A–D). The signal intensity of the amplified moose wild type isolate in moose wild type substrate was strongly reduced with P4 mAb, and the P4 epitope was completely lost in the two other moose substrates. In contrast, the moose Q_109_/Q_109_ isolate kept its P4 epitope in the Q_109_/Q_109_ and the K_109_/Q_109_ moose substrates (Figure 4A). All PMCA products of the red deer isolate lost the P4 epitope (Figures 4A–D).

## Discussion

In this study, we estimated conversion efficacies of five distinct Norwegian prion isolates using sPMCA. Two of the included isolates cause contagious diseases: one classical CWD isolate from reindeer and one from sheep with experimental classical scrapie. In addition, three isolates of sporadic prion diseases were included: one from red deer and two isolates from moose [16]. We found that prions from all isolates could be amplified in at least some of the brain substrates, and the isolates of the contagious diseases were the most efficient. The conversion efficacy was not only dependent on the origin of the isolate but also on the *PRNP* variant of the tested substrate.

Following the CWD outbreak in 2016, the entire reindeer herd in Nordfjella was culled, leaving the area reindeer-free [43], while the Hardangervidda plateau, the second affected area, still maintains a closely regulated reindeer population [44]. Both these areas are used as summer grazing pastures for sheep. Since concern exists for prion spillover, 500 sheep that had grazed in Nordfjella were investigated, and no prions were found in their gut-associated lymphoid tissue, despite habitat overlapping with diseased reindeer [31]. In experimental settings, classical North American CWD isolates overcame the species barrier when inoculated intracerebrally into scrapie susceptible sheep, albeit with a low transmission rate [37]. However, at the second passage, there was a 100% attack rate [45]. Inoculation by the oronasal route with a CWD isolate from white-tailed deer (WTD), resulted in transmission to only one of seven sheep carrying the scrapie susceptible A_136_R_154_Q_171_/A_136_R_154_Q_171_ genotype (wild type) after a long incubation time [46]. Based on these studies, sheep may have a low risk contracting North American CWD, but it is important to note that characterization of the Norwegian isolates so far indicate that these are different from the North American isolates [17, 18, 39].

One PMCA study that used substrates from transgenic ovinized mice carrying the A_136_R_154_Q_171_/A_136_R_154_Q_171_ genotype indicated that reindeer CWD has a relatively strong spillover potential towards sheep, but also the sporadic red deer isolate showed the same tendency, while the Norwegian moose CWD strain did not [18]. Interestingly, both the wild type moose isolate, and the red deer isolate characterized in that paper are the same as those utilized in the present study, but none of these isolates amplified in our three sheep substrates. We found weak conversion of the reindeer isolate in both scrapie susceptible A_136_R_154_Q_171_/A_136_R_154_Q_171_ and V_136_R_154_Q_171_/V_136_R_154_Q_171_ substrates, comparable to the amplification of a different Norwegian CWD affected reindeer in ovinized mice brain [18]. This could indicate a possibility for reindeer CWD spillover to sheep and in line with the North American studies (37, 46). Unexpectedly, the scrapie isolate was only weakly amplified in the goat substrate, which also appeared resistant to the cervid isolates. Several goat *PRNP* polymorphisms have been reported, and some genotypes have been associated with reduced susceptibility to classical scrapie. In this study, we utilized goat substrate with the Q_220_/L_220_ genotype, which does not appear to alter susceptibility to classical scrapie [47]. As expected, regardless of the isolate used, no conversion was observed in the scrapie resistant A_136_R_154_R_171_/A_136_R_154_R_171_ sheep substrate and the prion protein deprived Ter_32_/Ter_32_ goat substrate.

The newly emerging Norwegian reindeer CWD isolate presents a probable risk to other cervids, while the sporadic isolates are assumed to have a low risk of transmission since they are probably not contagious. Norway has substantial populations of wild cervids, encompassing four native species [30]. These wild cervids exhibit a well described spatial migration [48–50] and can potentially encounter environmental prions excreted by diseased reindeer. The reindeer isolate converted all four cervid species, and the experimental scrapie isolate had comparable amplification efficacy indicating that these two contagious diseases have a spillover potential to these species.

There are at least three different moose CWD isolates [15, 16, 39] in Fennoscandia and the disease appears so far in animals with either the wild type *PRNP*)K_109_/K_109_) or the Q_109_/Q_109_ variant. The two tested moose isolates in the present study were able to amplify all but one of the moose substrates. The wild type isolate also converted the wild type red deer substrate with identical *PRNP* sequence. When the same wild type isolate was tested against brain homogenates from transgenic mice expressing the deer (wild-type) or elk prion protein, both were amplified [18]. In yet another study, this isolate showed efficient transmission to Tg-mice expressing the cervid wild type *PRNP*, but slower and incomplete to Tg-E_226_ mice [39].

The European red deer population in Norway possesses only one *PRNP* polymorphism at position 226, in contrast to red deer in other European countries where multiple variants exist [51]. In North America, where European red deer have been introduced for recreational purposes, only a few cases of contagious CWD have been reported [52, 53]. Apart from these infrequent naturally occurring cases, four red deer with three different genotypes at position 226 were orally inoculated with a CWD isolate from Rocky Mountain elk. All animals developed clinical disease with detectable prions in their brains and lymphoid tissue at similar time point after inoculation, irrespective of the *PRNP* genotype [54]. The three sporadic CWD cases from Norway occurred in animals carrying the E_226_/E_226_ genotype [11, personal communication Sylvie L. Benestad]. Notably, the red deer isolate amplified in all cervid species, and with high efficacy in the moose substrate and the homologous red deer substrate. This contrasts with the other two sporadic isolates included in the study. In line with our results, the same red deer isolate had efficient amplification in two transgenic mice substrates with the wild type and E_226_/E_226_ variant *PRNP* [18]. Some of the results concerning the sporadic isolates may seem surprising and show that the behavior of these isolates under PMCA conditions may be less predictable than that of the contagious isolates.

Roe deer is the only species in our experiment with no reports of naturally occurring CWD, and to our knowledge, no inoculation studies have been performed in this species. Roe deer seems to maintain *PRNP* homology overall [51, 55, 56], and only one conference paper has reported a polymorphism in three roe deer in Portugal [57]. Interestingly, our study showed that three of the prion isolates amplified in the roe deer substrate, particularly the reindeer and scrapie isolates showing good conversion efficacy. These results suggest that attention should be given to this species concerning surveillance for CWD. In Norway, the roe deer population is quite large, estimated to be around 115 000 animals [30]. However, only a few animals were reported hunted in the municipalities around Nordfjella, and the spatial overlap of roe deer and reindeer is thought to be low [32]. While the roe deer population in Norway is part of the national surveillance program for CWD, it is noteworthy that the annual testing rate for these free-ranging cervids is the lowest compared to other species [58].

Norway is the only country in Western Europe with wild reindeer populations. Additionally, large populations of semi-domesticated reindeer can be found in the middle and north of the country. The reindeer exhibit a particularly diverse *PRNP* variation. The subspecies found in the Fennoscandian Peninsula, the Eurasian reindeer (*Rangifer tarandus tarandus*), was discovered to have seven non-synonymous base pair substitutions and one octapeptide deletion [56]. To date, 17 different genotypes have been identified among wild and semi domesticated reindeer in Norway. Contagious CWD in Norwegian reindeer has only been found in the wild type *PRNP* and in three different heterozygote variants. The wild type and the heterozygote wild type/octapeptide deletion variants seem to be equally susceptible to the disease [59]. The wild type *PRNP* is more common among wild reindeer than the semi-domesticated reindeer in Norway. Additionally, the octapeptide deletion is very rare among the semi-domesticated herds. Instead, these animals carry higher frequencies of genotypes which seem to be less susceptible to CWD [56]. The Eurasian reindeer has been successfully infected with North American CWD via the oral route [8, 9]. In one of the studies, reindeer were inoculated with CWD isolates from either elk or WTD, and only those that were inoculated with WTD CWD did accumulate prions in their brains and in peripheral organs. The *PRNP* of the inoculated animals was a variant not found in the Norwegian reindeer populations [8]. In our study, we discovered that both reindeer and experimental scrapie isolates induced conversion in the reindeer wild type substrate. Unexpectedly, the S_225_/Y_225_ substrate was not converted by any of the five isolates, despite several reported cases of natural CWD infection in this variant [59]. Furthermore, we observed no conversion activity in the Y_225_/Y_225,_ D_176_/D_176_, and M_2_S_129_M_169_/M_2_S_129_M_169_ reindeer substrates, which are genotypes not reported among the positive natural cases, when tested against the reindeer and moose wild type isolates, the only two isolates examined.

Finally, we used two monoclonal antibodies to further characterize the PMCA products and the PK cleavage sites of the isolates. We demonstrated loss or strong signal reduction of the N-terminal segment including the P4 epitope in the Norwegian moose CWD isolates across most substrates in accordance with an earlier study [10]. We noted that the amplified product of the previously uncharacterized Q_109_/Q_109_ isolate maintained the P4 epitope, as seen in the original isolate, in two of the moose substrates. Intriguingly, the rein deer isolate lost the P4 epitope when amplified in the same two moose substrates, while the scrapie and red deer retained their original signatures across all substrates. These findings indicate that, although, in most cases the cleavage site of PMCA products seems to be determined by the isolate itself, a molecular shift can be induced in the substrate that could possibly be explained by reduced compatibility between the seed and the substrate or some unknown characteristics of the isolate [60].

In these sPMCA experiments, we observed that prion isolates obtained from Norwegian reindeer can convert a range of substrates from different species with an efficacy comparable to that of an experimental classical scrapie isolate, suggesting a potential for spillover of these contagious diseases. Conversely, the conversion efficacy of isolates derived from moose and red deer, representing sporadic forms of CWD, was weaker than that of the contagious isolates. Still, three of the cervid isolates converted substrates of other cervid species suggesting a weaker species barrier and a transmission potential between these species. We also demonstrated that roe deer substrate was converted by three different CWD isolates, indicating that this species may be susceptible to prion disease and warrants closer attention.

## Data Availability

The datasets used and/or analyzed during the current study are available from the corresponding author on reasonable request.
